# Comparing Stacking Ensemble Techniques to Improve Musculoskeletal Fracture Image Classification

**DOI:** 10.3390/jimaging7060100

**Published:** 2021-06-21

**Authors:** Ibrahem Kandel, Mauro Castelli, Aleš Popovič

**Affiliations:** 1 Nova Information Management School (NOVA IMS), Universidade Nova de Lisboa, Campus de Campolide, 1070-312 Lisboa, Portugal; mcastelli@novaims.unl.pt (M.C.); apopovic@novaims.unl.pt (A.P.); 2 School of Economics and Business, University of Ljubljana, Kardeljeva Ploščad 17, 1000 Ljubljana, Slovenia

**Keywords:** deep learning, image classification, stacking, ensemble learning, convolutional neural networks, transfer learning, medical images

## Abstract

Bone fractures are among the main reasons for emergency room admittance and require a rapid response from doctors. Bone fractures can be severe and can lead to permanent disability if not treated correctly and rapidly. Using X-ray imaging in the emergency room to detect fractures is a challenging task that requires an experienced radiologist, a specialist who is not always available. The availability of an automatic tool for image classification can provide a second opinion for doctors operating in the emergency room and reduce the error rate in diagnosis. This study aims to increase the existing state-of-the-art convolutional neural networks’ performance by using various ensemble techniques. In this approach, different CNNs (Convolutional Neural Networks) are used to classify the images; rather than choosing the best one, a stacking ensemble provides a more reliable and robust classifier. The ensemble model outperforms the results of individual CNNs by an average of 10%.

## 1. Introduction

The incidence of bone fractures is affected by many factors including age, gender, biology, physiology, and access to treatment and prevention programs [1,2,3]. Impairment-related bone fractures contribute to an increase in morbidity and mortality across the age span [3]. The leading causes of bone fractures include osteoporosis [2,4,5] and trauma [6]. Osteoporosis is a chronic bone disease related to the loss of bone density. Bone trauma can be defined as an injury caused to the bone by a force external to the body. When applied to clinical practice, a systematic study of bone fracture data can allow clinicians to compare affected and unaffected patient groups, determine definable and preventable characteristics that predispose patients to skeletal fractures and ensure the provision of appropriate prevention and treatment strategies [7,8,9].

Deep learning is a subfield of artificial intelligence that has gained much attention due to its robust results in many challenging domains, such as machine translation, natural language processing, and computer vision, among others [10]. Convolutional neural networks (CNNs) are part of deep learning domains where at least one layer of the neural network is a convolution layer. Many state-of-the-art results were achieved by using CNNs, especially in the computer vision domain. However, one of the main drawbacks of using CNNs to classify images is the dataset size needed to train them accurately: thousands of images are usually required. This issue limits the usage of CNNs in classifying images in the medical field. However, two main techniques can be used to get over the issue of dataset size: transfer learning and ensemble learning. Transfer learning was introduced to get over the dataset size challenge by training a CNN on a sizeable nonmedical dataset, then fine-tuning the weights to a medical dataset. Ensemble learning is a technique based on the principle of the crowd’s wisdom. In ensemble learning, multiple classifiers are trained, and then their results are combined. There are different methods of combining the outputs of different classifiers, such as taking the average of their outputs or using a machine learning algorithm over their predictions—that is, creating a classifier to sort different models’ outputs.

The classification of fracture images using CNNs was investigated in many studies. Chung et al. [11] used a private dataset to investigate shoulder image usage to classify humerus fractures using ResNet CNN. In particular, they used a dataset with 256 × 256-pixel images. After fine-tuning the CNN, they applied the CNN model to reach an accuracy of 96%, a performance higher than that of human experts. Rajpurkar et al. [12] investigated CNN’s performance in classifying bone fractures using a novel dataset called MURA. The images were rescaled to 320 × 320 pixels, and they compared the performance of their networks to three different radiologists’ assessments. The radiologists’ performance was more accurate than their results.

Olczak et al. [13] tested CNN on a private dataset with 256,000 images split among wrist, hand, and ankle images. The X-ray images were rescaled to 256 × 256 pixels, and VGG16 achieved the best performance. Additionally, CNN’s performance was comparable to two senior orthopedic surgeons. Lindsey et al. [14] studied the effect of having a CNN to classify wrist fractures in the ER to help in fracture diagnosis. They used a private dataset to train a CNN by relying on an extension of U-Net architecture as a classifier. Subsequently, they did a controlled experiment to evaluate the importance of having a CNN to aid doctors’ diagnoses. Olczak et al. [13] found a significant decrease of 47% in the misinterpretation rate when using CNN.

Uysal et al. [15] studied the effect of different SOTA CNN architectures on the MURA dataset’s shoulder images. The images were scaled to 320 × 320 pixels. The Adam optimizer was chosen, with a learning rate of 0.0001 with 40 epochs for training. The best kappa score was 0.6942, resulting from taking an ensemble of ResNet34, DenseNet169, DenseNet201, and a sub-ensemble of different CNNs. 

Guan et al. [16] used 3392 positive images (images with fractures) of the humerus, elbows, finger, hand, and forearm fracture from the MURA dataset to detect bone fractures. The image size was scaled to be 800 pixels for the shorter side and 1333 pixels for the longer side. Subsequently, an improved two-stage RCNN detection method was proposed. However, the original MURA dataset did not contain annotations of exact fracture locations. For this reason, the locations were annotated using the help of three radiological experts with more than 20 years of experience. To mitigate the small dataset’s effect, two main geometric augmentation techniques were considered: horizontal flipping and random rotation. The authors used 4 GPU NVIDIA GeForce GTX 1080Ti graphic cards to train the model. The average precision reported in the study was 62.04% [16]. 

Huynh et al. [17] used the humerus images from the MURA dataset. The images were rescaled to 150 × 150 pixels, and a novel CNN architecture with two convolution layers and one max pooling layer was presented. The learning rate of 0.0001 achieved the best results compared to three different learning rates. Moreover, the proposed model achieved a validation accuracy of 0.684 compared to a kappa score of 0.600 for the MURA dataset. Urinbayev et al. [18] used the MURA dataset as a preprocessing step to determine the organ type before further classification. Thus, the dataset was used in the context of organ type classification and not for fracture classifications. The main aim was to classify chest X-ray images. 

Kitamura et al. [19] investigated the performance of three SOTA CNN models on a private dataset. The dataset used was composed of 596 ankle images. The image size was 300 × 300 pixels. Four different geometric augmentation techniques were used to increase the size of the training dataset and to make the models more invariant. The techniques used were random rotation, flipping, brightness variation, and contrast alteration. An NVIDIA GeForce 1080 GTX graphics card was used to perform the experiments, in which the performance of each of the three CNNs and an ensemble composed of the three models were investigated. The performance of the ensemble methods was better than any single model’s performance, and the best accuracy recorded was 81%.

Many studies have investigated the effects of ensemble techniques, especially their impacts on CNNs. Chouhan et al. [20] proposed a study to classify pneumonia images and used five different CNNs. By taking the average of the five CNNs instead of using them individually, it was possible to increase the final model’s accuracy. A 2% increase in accuracy was achieved by using the average instead of using the best-performing CNN. Even with the robust model introduced by He et al. [21] in 2016, the authors used the ensemble technique to win the ImageNet challenge [22]. Whereas their top model scored a 4.49% error rate, using ensemble techniques allowed them to reduce the error rate to 3.57%. Rajaraman et al. [23] investigated four different approaches to accurately classify tuberculosis from X-ray images. They reported that stacking achieved the best results. Cha et al. [24] studied different techniques for classifying ear diseases by using otoendoscopic images. They compared the performance of nine CNNs and then selected the best two CNNs to build an ensemble model. The ensemble model achieved the best result compared to the other CNNs taken into account. Kandel and Castelli [25] investigated the impact of transfer learning, i.e., using the weights of ImageNet instead of training the network from scratch on X-ray classification to detect fractures. They used the seven MURA datasets [12], and investigated six state-of-the-art CNN architectures: VGG19 [26], InceptionV3 [27], ResNet50 [21], DenseNet [28], Xception [29], and InceptionResNet [30]. Experimental results show that the weights of ImageNet had an impact other than training the CNN from scratch. Moreover, sometimes, by training the networks from scratch, it did not converge at all, given the MURA dataset’s limited size [12]. 

Joshi et al. [31] reviewed several contributions that apply artificial intelligence techniques to fracture detection. Among techniques, they pointed out that only a few works use CNNs for fracture detection. They cited five papers in which CNNs were used with data augmentation for fracture detection. While their work suggests that stacking was used in the past with traditional (fully connected) neural networks, random forests, and support vector machines, no study was cited on the use of stacking with CNNs. Thus, we believe our contribution is timely and provides an interesting contribution to this field.

Our primary goal is to develop a classification framework for bone fracture detection using X-ray images. To reach our goal, we compared different stacking techniques to improve the classification of fractures using X-ray images. 

The main contributions of this work are as follows:

(1) We evaluate different state-of-the-art CNNs. We also assess their combinations’ performance, either in averaging, weighted average, or majority vote.
(2) We investigate the usage of the stacking ensemble along with CNN outputs. To do so, we tested the performance of eight different machine learning meta-classifiers. Two main methods are considered: using probability outputs to construct the level-1 ensemble, or using label outputs.

The rest of this paper is organized as follows: In the second section, we describe our methodology. The third section presents our results obtained. The fourth section discusses the main findings of this study. Finally, the fifth section concludes the paper.

## 2. Materials and Methods

**Figure 1:**
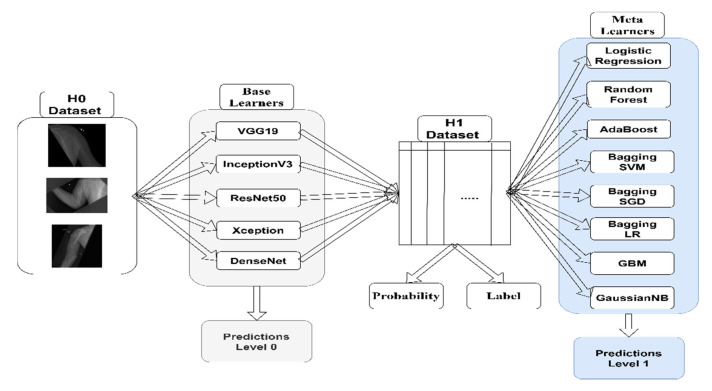
Schematic diagram of the proposed pipeline.

In Figure 1, we illustrate a schematic diagram of the proposed pipeline, showing how we stacked different machine learning algorithms. In the following subsections, we briefly discuss the methods used in this research and the dataset used to train the CNN.

### 2.1. Conventional Neural Networks

After CNN’s success in the ImageNet Large Scale Visual Recognition Challenge (ILSVRC challenge), it became the de facto algorithm for image classification. The difference between CNNs and any other neural network is the presence of the convolution layer. The convolution layer’s importance is that it decreases the number of connections required and considers the spatial and temporal information of images. The convolution layer works by applying a window size called the kernel that convolves the image to detect essential features that can be used in the classification task. Equation (1) shows the convolution operation for colored images:

(1) $$O\left[i,j\right]=F\left(u,v\right)*I\left(i,j\right)=\sum_{u}\sum_{v}\sum_{c\in \left\{R,G,B\right\}}F_{c}\left(u,v\right)\;⊙\;I_{c}\left(i+u,j+v\right)$$

where I(.) is the input image, c is the color channels, F(.) is the kernel, and O[i,j] is the output pixel in the (i,j) position. Moreover, the asterisk * operation resembles the convolution operation between the image and the kernel, which equals the dot product’s summation ⊙ between different pixels in the (i,j) position.

A CNN mainly consists of two types of layers: primary layers and secondary layers. Primary layers are mandatory layers that constitute the CNN (i.e., convolution layers, pooling layers, and fully connected layers). Secondary layers (e.g., dropout layers and normalization layers) are used to increase CNN performance.

#### 2.1.1. VGG19 Network

VGG networks were introduced by Simonyan and Zisserman [26] to participate in the ImageNet challenge. VGG networks are composed of sequential convolutional blocks separated by max-pooling layers. VGG networks come in many variants. We used a 19-layered version (i.e., VGG19). 

#### 2.1.2. InceptionV3 Network

The InceptionV3 network was introduced by Szegedy et al. [32] to participate in the ImageNet challenge. The InceptionV3 network has a novel module called the inception module, where convolution layers are connected in parallel and sequentially. 

#### 2.1.3. Resnet Network

ResNet was introduced by He et al. [21] in 2015. They proposed a new connection called the residual connection to minimize the vanishing gradient problem that usually happens to very deep networks (i.e., networks with more than 30 layers).

#### 2.1.4. Xception Network

The Xception network was introduced by Chollet [29]. They used depth-wise separable convolutional layers instead of conventional convolutional layers.

#### 2.1.5. Densenet Network

DenseNet was introduced by Huang et al. [28]. They were inspired by the residual connection proposed in the ResNet CNN [21]. Instead of using the residual connection, they densely connected the convolutional layers. In addition, the layers were concatenated instead of added to each other, similar to ResNet.

### 2.2. Machine Learning Algorithms

In this section, we provide a summary of the different machine learning algorithms used in this paper. 

#### 2.2.1. Logistic Regression

This is a simple linear model with a binary outcome. Logistic regression models the target variable with a line for two dependent variables or a hyperplane for more than two variables. Equation (2) shows the logistic operation:

(2) $$p=\frac{1}{1+e^{-\left(\beta_{0}+\beta_{1}x_{1}+\ldots +\beta_{n}x_{n}\right)}}$$

where p is the probability of success (in our study is the presence of fracture), $\beta_{0}$ is the model intercept, and $\beta_{i}$ are the regression coefficients. 

#### 2.2.2. Bagging and Random Forests

Breiman [33] introduced the bagging algorithm to decrease the high variance of decision trees. “Bagging” stands for bootstrap aggregation, where several decision trees are constructed using sampling with replacement from the dataset. The trees’ outputs are averaged in case of regression, and a majority vote is taken in the event of a classification task. Breiman introduced an upgrade to bagging called random forests [34]: to decorrelate the trees, only a subset of the variables is randomly selected in each step to construct a decision tree. Bagging and random forests consider homogenous learners. 

#### 2.2.3. AdaBoost

AdaBoost (adaptive boosting) [35] is an ensemble approach that uses an iterative process. The main idea is to give more importance to misclassified data points built by the first weak learner, then build another weak learner that concentrates on these misclassified data points. This iterative nature of the algorithm ensures that each misclassified data point will have a learner specifically built to focus on it. The formula of AdaBoost is presented in Equation (3):

(3) $$F\left(x\right)=sign\left(\sum_{k=1}^{K}\theta_{k}f_{k}\left(x\right)\right)\;$$

where K is the number of weak classifiers, $\theta_{k}$ is the weight of the Kth weak classifier, and $f_{k}\left(x\right)$ is the Kth weak classifier. 

#### 2.2.4. Gradient Boosting Machine

A gradient boosting machine [36] is an iterative ensemble technique. Usually, the weak learners are decision trees. The weak learners have residuals that the subsequent learner tries to minimize. In the stochastic gradient descent algorithm, Friedman [37] proposed building weak learners on samples drawn from a dataset by using sampling with replacement technique to give the model some variability and decrease bias.

#### 2.2.5. Naïve Bayes

Naïve Bayes (NB) is a classifier based on the Bayes theorem, which is calculated by estimating probabilities. The NB classifier assumes that every conditional probability of each feature is independent of each other. According to Bayes’ theorem, NB can be calculated using Equation (4):

(4) $$P\left(y|x\right)=\frac{P\left(x|y\right)P\left(y\right)}{P\left(x\right)}$$

where P(x|y) is the conditional probability of each feature, P(x) is the feature’s prior probability, and P(y) is the target class y’s prior probability. Generally speaking, NB classifiers have three main subclasses: Gaussian, multinomial, and Bernoulli. In this study, we used the GaussianNB classifier, in which the likelihood of features is assumed to be Gaussian. This can be calculated using Equation (5):

(5) $$P\left(x|y\right)=\frac{1}{\sqrt{2\pi \sigma_{y}^{2}}}e^{\left(-\frac{{\left(x-\mu_{y}\right)}^{2}}{2\sigma_{y}^{2}}\right)}$$

where the parameters $\sigma_{y}^{2}$ and $\mu_{y}$ are the variance and the mean, respectively. These values are estimated using maximum likelihood. For more information, the reader is referred to the corresponding article [38]. 

### 2.3. Stacking

**Figure 2:**
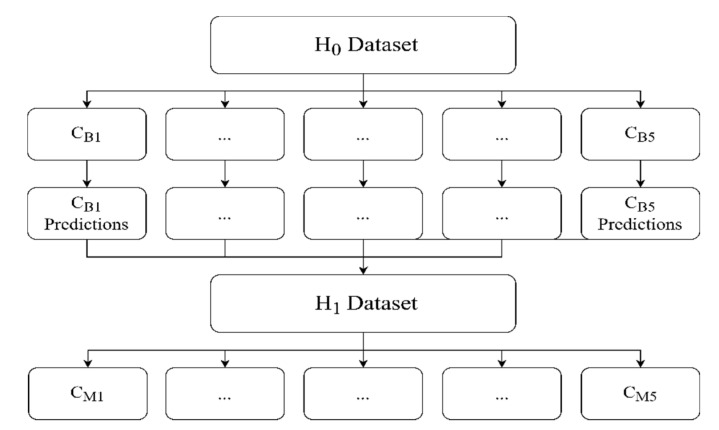
A schematic diagram of the stacking method.

Wolpert [39] introduced stacking as an ensemble algorithm that is different from bagging, random forest, and boosting: stacking considers heterogeneous learners. A schematic diagram of the stacking method is presented in Figure 2. There are usually two or more levels of classifiers. The first level is called the zero level, and it contains the base classifiers that take the original inputs. As seen in Figure 2, $H_{0}$ is the original dataset, which is the MURA dataset in our case. The zero-level classifiers will produce the $H_{1}$ dataset, which will be used in the second level by the meta-classifiers (or level-one classifiers). $H_{1}$ is the dataset that will be produced by the base classifiers, which are the CNNs in our case. $C_{B_{i}}$ are the base classifiers that will be used to produce the $H_{1}$ dataset. $C_{M_{i}}$ are the meta-classifiers that will be used for classification of the $H_{1}$ dataset. $H_{1}$ could be probabilities or labels, meaning the output of the $C_{B_{i}}$ that will be used by $C_{M_{i}}$. We will compare both methods.

### 2.4. Evaluation Metrics

Two evaluation metrics were used to assess each classifier’s performance. Below are the summaries for each.

#### 2.4.1. Accuracy

The percentage of correctly classified images to the total number of images. Can be calculated using Equation (6):

(6) $$Accuracy=\frac{TP+TN}{TP+TN+FP+FN}$$

where *TP* is the number of true positive images, *TN* is the number of truly negative images, *FP* is the number of false-positive images, and *FN* is the number of false-negative images. 

#### 2.4.2. Kappa Score

The Kappa score [40] measures the agreement between the actual label and the predicted label. It ranges from −1 to +1, where +1 means that the classifier predicts the correct labels and ≤0 means that the classifier is just a random guess. The Kappa score can be calculated using Equation (7):

(7) $$Kappa=\frac{Agreement_{Observed}-Agreement_{Expected}}{1-Agreement_{Expected}}\;\;\;$$

where $Agreement_{Observed}$ is the accuracy of classifier C and $Agreement_{Expected}$ is calculated using Equation (8).

(8) $$Agreement_{Expected}=\frac{1}{N^{2}}\sum_{k}n_{k1}n_{k2}$$

For k mutually exclusive categories with N total data points and $n_{ki}$ the number of times classifier C predicted category k.

### 2.5. Dataset

**Figure 3:**
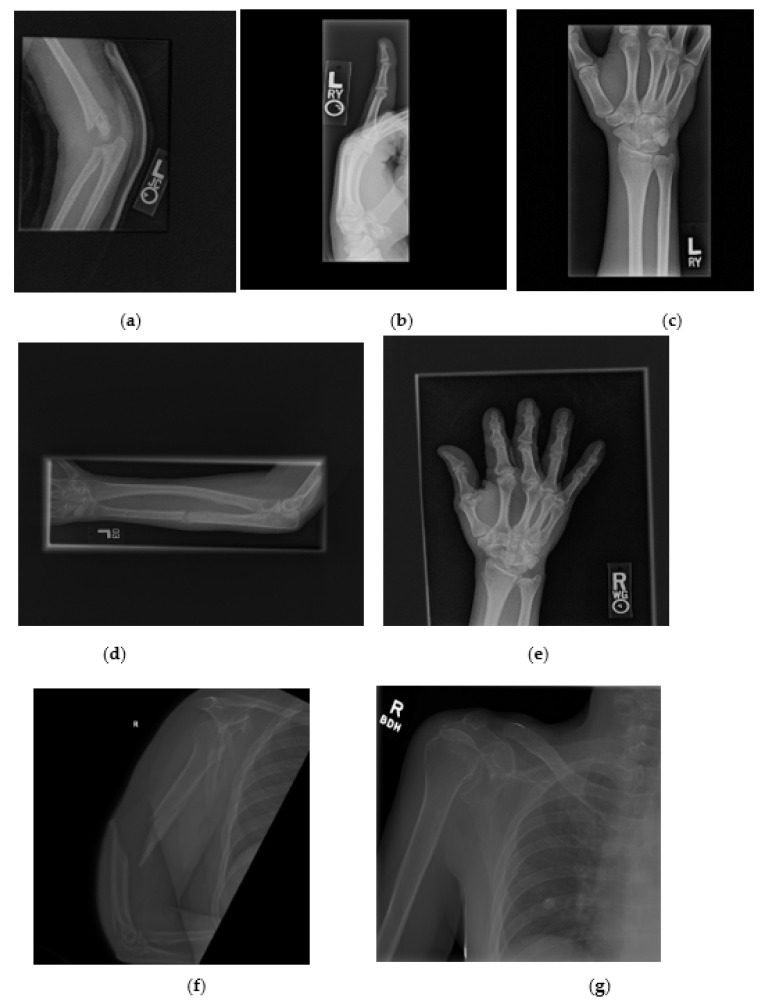
Samples of each fracture type from MURA dataset: (**a**) Fractured elbow. (**b**) Fractured finger. (**c**) Fractured wrist. (**d**) Fractured forearm. (**e**) Fractured hand. (**f**) Fractured humerus. (**g**) Fractured shoulder.

The dataset used in this paper is the publicly available MURA dataset introduced by Rajpurkar et al. [12]. MURA includes seven different musculoskeletal categories, namely wrist, hand, elbow, shoulder, forearm, finger, and humerus. The dataset contains 40,005 images split into 92% for training and 8% for testing. The original size of the images is not constant and ranges from 512 × 512 pixels to 97 × 512 pixels. The file extension of the images is .png. In Figure 3, a sample of each fracture type is shown.

## 3. Results

As pointed out by [19,41], fine-tuning the CNN architectures will yield better results and converge faster than training a CNN from scratch. In this paper, we fine-tuned all CNN layers using the ImageNet dataset. All hyperparameters were fixed for all CNNs during the training process. We used the Adam optimizer [42] with a learning rate of 0.0001 for all CNNs. The batch size was 64. All images were rescaled to 96 × 96 pixels. An early stopping criterion was used to stop the training if no progress occurred for 50 epochs. Four image augmentation techniques were considered to increase the model performance: zooming, 180° rotations, and horizontal and vertical flips. We relied on data augmentation to balance the numbers of images in the different classes. All machine learning algorithms’ hyperparameters were kept at the default settings of the Scikit-learn Python package. An NVIDIA RTX 2060 graphics card and an Intel i7-10750H CPU were used for training.

Due to the dataset’s size, we did not use point estimates. Instead, each base-learner classifier was trained 10 times, then average scores were calculated. To determine the statistical significance of the results, we followed the methods of [43,44], in which a small confidence interval implies statistical significance [45]. For the sake of readability, we provided a summary of the results for all images in Table 1. Full tables may be found in the annex. 

In the first experiment, we tested different techniques on the humerus images. The first set of experiments tested the performance of various state-of-the-art CNNs on the humerus dataset, which considered level-0 classifiers. The VGG19 architecture achieved the highest score among the five CNNs, κ = 0.6299 ± 2.40%. This was the greatest kappa value and the smallest confidence interval compared to other level-0 classifiers. The ResNet50 network achieved the lowest score, κ = 0.5349 ± 3.67% but had the greatest confidence interval. Subsequently, to assess the performance of statistical metrics, we averaged the predictions of the five CNNs. A majority vote was taken, and the weighted average based on kappa scores was calculated. The three scores were approximately the same, with the average score being slightly higher than the others. The second set of experiments aimed at training a machine learning classifier over the probability outputs of the CNN. The NB classifier achieved the highest score, with a score slightly lower than the one achieved in the first set of experiments. The third set of experiments was similar to the second but considered the label outputs of the CNN. The scores achieved in the third experiment were smaller than the ones achieved in the first and the second sets of experiments. Overall, the highest score was achieved by taking the average of the level-0 classifiers for the humerus images. 

We tested the different techniques on the finger fracture images, as we had with the humerus images. The first set of experiments considered the level-0 classifiers. The best CNN was the DenseNet network, κ = 0.4168 ± 3.54%; while the InceptionV3 network achieved the lowest score, κ = 0.3566 ± 2.41%. To assess the performance of simple statistical metrics, we averaged the predictions of the five CNNs. A majority vote was taken, and the weighted average based on kappa scores was calculated. The kappa score of the average vote and the weighted vote were the same and were higher than the majority vote and the DenseNet score from level 0. Overall, in the first set of experiments, the highest score was achieved by considering the CNNs’ average vote. In the second set of experiments, each CNN’s probability score was used to train a machine learning classifier. The highest score was achieved by the NB classifier, κ = 0.4862 ± 2.42%. The score achieved by the NB classifier was greater than all previous scores. For the third set of experiments, the NB classifier also achieved the highest score; however, it was less than that achieved by using the probability score. Overall, the second set of experiments’ performance was better than both the first and the third sets of experiments. 

In the first set of experiments for elbow images, the greatest kappa scores were achieved by the VGG19 network, κ = 0.6436 ± 2.19%; and the Xception network, κ = 0.6433 ± 0.91%. The VGG19 network’s kappa value was slightly high; however, the Xception network’s confidence interval was less than that of the VGG19 network. To assess the performance of simple statistical metrics, the predictions of the five CNNs were averaged, the majority vote was taken, and the weighted average based on kappa scores was calculated. All three kappa scores were similar to the weighted average score (0.6970 ± 0.67%), which was slightly greater than the others with the smallest confidence interval. In the second set of experiments, the probabilities of the CNNs were used to train eight machine-learning classifiers. All scores were approximately similar, with the LR bagging classifier being the highest, κ = 0.6758 ± 1.74%. Overall, the scores of the second set of experiments were lesser than those of the first set of experiments. The third set of experiments’ results were lesser than those achieved in the first and second experimental sets. AdaBoost classifier scored the highest, κ = 0.6720 ± 1.78%. Overall, the best kappa score achieved for the elbow images was achieved by taking the weighted average vote of level-0 classifiers. 

For wrist images, the best score achieved by a level-0 classifier was by the Xception network, κ = 0.6127% ± 1.59%. To assess the performance of simple statistical metrics, the predictions of the five CNNs were averaged, the majority vote was taken, and the weighted average based on kappa scores was calculated. The highest score was achieved by the weighted average of the level-0 classifiers, κ = 0.6556 ± 0.58%, which was greater than the Xception network’s score. In the second set of experiments, the best performer was the logistic regression classifier, κ = 0.6510 ± 1.06%. While this score was greater than the level-0 classifiers’ scores, it was still less than the weighted average vote of the level-0 classifiers. The results of the third set of experiments were lesser than both the first and second sets. Overall, in all experiments, the weighted average vote of the level-0 classifiers yielded the best results for wrist images. 

For forearm images, the best CNN was DenseNet, κ = 0.5592 ± 2.16%. To assess the performance of simple statistical metrics, the predictions of the five CNNs were averaged, the majority vote was taken, and the weighted average based on kappa scores was calculated. The weighted average votes achieved the highest score, κ = 0.5765 ± 1.85%. The results achieved in the second set of experiments were better than those of the first set, with the NB classifier achieving the highest kappa score, κ = 0.6195 ± 2%. The results achieved in the third set of experiments were as high as the second set, with the NB classifier achieving the highest kappa score as well, κ = 0.6201 ± 1.76%. Overall, the NB classifier using label predictions achieved the best results for forearm images. 

For hand images, the VGG19 network achieved the highest score among all CNNs, κ = 0.4358 ± 1.68%. To assess the performance of simple statistical metrics, the predictions of the five CNNs were averaged, the majority vote was taken, and the weighted average based on kappa scores was calculated. The weighted average votes achieved the highest score, κ = 0.4260 ± 2.30%. However, this score was less than the score achieved by the VGG19 network. The results achieved in the second set of experiments were better than those of the first set, with the NB classifier achieving the highest kappa score, κ = 0.5029 ± 1.14%. The results achieved in the third set of experiments were slightly lesser than the second set, with the NB classifier achieving the highest kappa score, κ = 0.4962 ± 2.62%). Overall, the best result was achieved by an NB classifier using probabilities. 

For shoulder images, the best performing CNN was the VGG19 network, κ = 0.4638 ± 2.49%. To assess the performance of simple statistical metrics, the predictions of the five CNNs were averaged, the majority vote was taken, and the weighted average based on kappa scores was calculated. The weighted average votes achieved the highest score, κ = 0.4908 ± 2.19%. For the second set of experiments, the highest score was achieved by the GBM classifier, κ = 0.5050 ± 2.09%. The GBM kappa score was greater than both the level-0 classifiers and their average weight score. For the third set of experiments, the bagging classifier achieved the highest score using logistic regression, κ = 0.4882 ± 2.30%. Overall, the best classifier for shoulder images was the GBM classifier trained using probability scores. Appendix A contains all the results for the expriments we performed.

## 4. Discussion

In this study, rather than relying on a single CNN classification, we investigated different methods to combine the results of individual CNN networks (level-0 classifiers) in order to classify musculoskeletal X-ray images. We first trained five different CNN networks, then assessed their performance. Afterward, we combined their predictions by taking an average of their votes, a weighted average of their votes, or by taking their majority votes. We called this last step a meta-learner using statistics. Afterward, we examined the eight different machine learning algorithms’ performance on top of the predictions made by level-0 classifiers. Two different inputs were used to train the machine learning algorithms—either the probability output of the level-0 classifiers or their label output. In Table 2, we present the differences between the kappa scores of the best performing level-0 classifiers (CNNs) and those of the level-1 classifiers (machine learning algorithms). 

By comparing the meta-learners using statistics (MLUSs) across the seven different datasets, we observed that their performance was usually better than any single CNN, except for in the hand dataset, whose best-performing CNN (VGG19) achieved a better score than the three combination methods. Across the three methods, the weighted average achieved the best results. Even in the case of the hand dataset, it yielded a better score. It is worth noting that the weights were measured based on kappa scores. The greatest difference between the meta-learners using statistics and the best CNN occurred in the elbow dataset, with an average difference of 8.30%. The least difference occurred in the hand dataset, where the best CNN achieved better results than the combinations by an average of 5.06%.

Comparing the meta-learners using probability (MLUPs) to level 0, we observed that the MLUP classifiers achieved better results than the level-0 CNNs. The most significant difference was spotted in the finger dataset, where the average increase was 12.36% compared to level 0. The least difference occurred in the humerus dataset, in which the average increase was 2.68% compared to level 0. Additionally, across the eight machine learning algorithms, all achieved more accurate results than the best level-0 classifier. The best achieving meta-classifier was NB for four datasets. Comparing MLUPs to MLUSs, MLUPs achieved higher results than MLUSs for four datasets, and MLUSs achieved the best results for the remaining three datasets. However, it is worth noting that for the hand dataset, where the score of the best level-0 classifier achieved better results than all the MLUSs, MLUPs achieved significantly better results than the MLUSs, with an average 9.59% increase in accuracy. 

Comparing the meta-learners using labels (MLULs) to level-0 CNNs, we observed that the MLUL meta-classifiers achieved better results than all level-0 CNNs. The most significant difference was in the finger dataset, where the average increase was 8.78% compared to level 0. The least difference occurred in the humerus dataset, where the average increase was 0.61% compared to level 0. However, across the eight machine learning algorithms, some scored lower than the highest level-0 classifiers, such as the SGD bagging classifier for the humerus and shoulder datasets and the SVC bagging classifier for the humerus dataset. The best meta-classifier was the NB classifier, as it achieved the best results five times. Comparing MLULs to MLUSs, MLUSs achieved higher results than MLULs for four datasets. The MLUSs achieved the best results for the remaining three datasets. However, it is worth noting that for the hand dataset, where the score of the best level-0 classifier achieved better results than all MLUSs, MLULs achieved significantly better results than the MLUSs, with an average increase of 7.66%. Comparing MLUPs to MLULs, MLUPs achieved better results than MLULs for all seven datasets. 

From our results, it is clear that using stacking achieves better results than using any single classifier on its own. This conclusion matches the results obtained by several works presented in the literature [46]. One of the leading hypotheses for why stacking achieves better results than any single classifier is that combining the outputs of different classifiers can decrease each classifier’s error. This diversity is what makes stacking work [47,48], whereas the underlying level-0 classifiers must be different from each other and must make different errors. In our case, each CNN was different from the others both in its number of parameters and its underlying architecture, so each CNN made different errors.

Regarding the computational power needed for each MLUS, MLUP, and MLUL, the least was needed for the MLUSs. Here, no high computational power was needed since the method is all about taking the average or the majority vote. However, for MLUPs and MLULs, greater computational power was needed to train the machine learning algorithms. Nevertheless, it is worth noting that the computational power needed for the machine learning algorithm is less than the power needed to train CNNs by order of magnitude. Full tables on computational requirements can be found in the annex. 

Our choice of dataset was successful for two reasons. First, the dataset has seven different types of images. Second, each of the seven datasets has a different size. Thus, it was possible to compare our results across different types of images and different dataset sizes. Based on our results, we conclude that stacking algorithms achieve higher results than level-0 algorithms alone. MLUPs achieved better results than MLULs in all datasets. Thus, we recommend using MLUPs over MLULs. However, further studies are needed to compare MLUSs to MLUPs and determine which method is better.

Concerning other results presented in the literature, Kitamura et al. [19] investigated an ensemble method’s performance built on three CNNs: InceptionV3, ResNet, and Xception. They reported that using an ensemble (where the voting method was used to combine the different CNNs’ outputs) achieved better results than using a single model. Moreover, they investigated combining all the models versus combining only the best-achieving models. The performance of the ensemble using all models was better than that of the ensemble built on the best models only [19]. Both our study and Kitamura et al.’s [19] concluded that an ensemble of different CNNs would yield better results than using a single CNN.

Chung et al. [11] used a single model (ResNet) to classify humerus fracture images. They compared the performance of the single model to human experts. However, they neither compared their model to other CNN models nor examined any ensemble models’ performance. Olczak et al. [13] compared five different CNN architectures and selected the best model, which was the VGG16 network. They did not examine the performance of any ensemble models. Similarly, Lindsey et al. [14] did not investigate the role of ensemble learning and instead relied on simple learners. 

The main difference between our study and Uysal et al.’s [15] is that we used all seven different MURA datasets. Uysal et al. [15] used only the shoulder dataset. Thus, their results cannot be generalized over the different types of fracture images. Additionally, compared to Uysal et al. [15], we repeated each experiment 10 times to increase the significance of our findings. On the other hand, Uysal et al. [15] provided no average results and only presented their findings on a single model. They reported a kappa score exceeding our highest score for the shoulder dataset; we might speculate that this is due to the fact that they considered high-resolution images. All in all, considering the different experimental settings between our work and Uysal et al.’s work, the results obtained for the shoulder dataset are comparable.

The main difference between our study and Huynh et al.’s [17] is that we used all seven MURA datasets, while Huynh et al. [17] used only the humerus dataset. Thus, in this case as well, it is difficult to generalize their findings on all the different types of fracture images. The authors compared their model’s validation accuracy score to the MURA study’s kappa score [12]. Our score was comparable to that reported by Huynh et al. [17], even if our image sizes were smaller than the sizes they considered. Our score for the humerus dataset was κ = 0.6662 ± 1.60%, compared to κ = 0.684 reported by [17].

## 5. Conclusions

Using deep learning techniques in the emergency room can be very helpful for doctors when detecting fractures. In particular, deep learning models can reduce the time needed to classify fracture types. In this study, we discussed several ensemble techniques that can be used to improve musculoskeletal fracture classification performance. In the first set of experiments, we averaged the predictions of five CNNs, calculated a weighted average, and took a majority vote among CNNs to assess the performance of simple statistical metrics. In the second set of experiments, we investigated different machine learning algorithms as meta-classifiers for stacking techniques trained on the probability output of level-0 CNNs. In the third set of experiments, the meta-classifiers were trained on the level-0 CNNs’ label output. From our results, we conclude that using stacking algorithms achieves better results than using a single CNN.

However, MLUSs achieved better results than level-0 classifiers in six out of seven datasets. MLUSs have no high computational power requirements. MLUPs and MLULs achieved better results than level-0 classifiers in all datasets but required greater computational power. MLUPs achieved better accuracy than MLULs, so we conclude that MLUPs are better than MLULs. Nevertheless, the question of whether MLUPs or MLUSs are more accurate still requires further investigation. 

## Figures and Tables

**Table 1:** The average Kappa score achieved by each classifier for every organ (±Confidence Interval 95%). Test performance is reported.

Classifier	Humerus	Finger	Elbow	Wrist	Forearm	Hand	Shoulder
Base-Learner							
VGG19	0.630 ± 2.40%	0.371 ± 4.83%	0.644 ± 2.19%	0.600 ± 0.99%	0.508 ± 2.55%	0.436 ± 1.68%	0.464 ± 2.49%
InceptionV3	0.592 ± 2.71%	0.357 ± 2.41%	0.612 ± 2.25%	0.591 ± 2.16%	0.503 ± 4.36%	0.347 ± 3.01%	0.389 ± 4.35%
ResNet50	0.535 ± 3.67%	0.361 ± 3.62%	0.624 ± 2.16%	0.583 ± 2.39%	0.543 ± 3.64%	0.360 ± 4.84%	0.415 ± 4.53%
Xception	0.605 ± 2.56%	0.405 ± 1.68%	0.643 ± 0.91%	0.613 ± 1.59%	0.527 ± 3.11%	0.394 ± 4.39%	0.421 ± 3.43%
DenseNet	0.573 ± 2.50%	0.417 ± 3.54%	0.639 ± 2.10%	0.598 ± 1.54%	0.559 ± 2.16%	0.381 ± 4.06%	0.453 ± 2.73%
Meta-Learner using Statistics							
Average	0.666 ± 1.60%	0.440 ± 2.01%	0.696 ± 0.73%	0.655 ± 0.72%	0.572 ± 2.04%	0.419 ± 2.60%	0.488 ± 2.17%
Majority Vote	0.662 ± 1.67%	0.424 ± 1.68%	0.691 ± 0.94%	0.647 ± 0.86%	0.563 ± 2.18%	0.414 ± 2.63%	0.485 ± 2.57%
Weighted Average	0.666 ± 1.69%	0.440 ± 1.92%	0.697 ± 0.67%	0.656 ± 0.58%	0.577 ± 1.85%	0.426 ± 2.30%	0.491 ± 2.19%
Meta-Learner using probability							
Logistic Regression	0.651 ± 1.33%	0.473 ± 1.83%	0.675 ± 1.71%	0.651 ± 1.06%	0.600 ± 1.76%	0.482 ± 1.56%	0.489 ± 2.63%
Radom Forest	0.643 ± 1.40%	0.466 ± 2.08%	0.665 ± 2.04%	0.626 ± 1.37%	0.597 ± 2.38%	0.468 ± 2.00%	0.489 ± 1.64%
AdaBoost	0.631 ± 1.60%	0.465 ± 0.98%	0.664 ± 1.64%	0.641 ± 1.33%	0.579 ± 1.91%	0.474 ± 0.99%	0.498 ± 2.56%
Bagging Classifier SVC	0.645 ± 1.44%	0.454 ± 1.55%	0.674 ± 1.49%	0.650 ± 0.85%	0.590 ± 2.64%	0.460 ± 1.93%	0.505 ± 2.42%
Bagging Classifier SGD	0.648 ± 1.09%	0.455 ± 1.81%	0.674 ± 1.97%	0.644 ± 1.16%	0.610 ± 1.96%	0.466 ± 2.58%	0.490 ± 3.58%
Bagging Classifier LR	0.648 ± 1.17%	0.472 ± 1.72%	0.676 ± 1.74%	0.650 ± 1.04%	0.600 ± 2.08%	0.482 ± 1.52%	0.486 ± 2.58%
GBM	0.647 ± 1.59%	0.475 ± 1.52%	0.674 ± 1.63%	0.644 ± 1.12%	0.596 ± 2.28%	0.487 ± 1.86%	0.505 ± 2.09%
Gaussian NB	0.661 ± 1.28%	0.486 ± 2.42%	0.673 ± 1.58%	0.642 ± 1.04%	0.620 ± 2.00%	0.503 ± 1.14%	0.501 ± 1.75%

**Table 2:** Difference between the percentage values of the Kappa score of the highest level-0 classifiers (CNNs) and the Kappa score of the level-1 classifiers (machine learning algorithms).

	Humerus	Finger	Elbow	Wrist	Forearm	Hand	Shoulder
Reference	VGG19	DenseNet	VGG19	Xception	DenseNet	VGG19	VGG19
Meta-Learner using Statistics							
Average	5.77%	5.60%	8.17%	6.90%	2.28%	−3.85%	5.31%
Majority Vote	5.12%	1.70%	7.36%	5.58%	0.73%	−5.06%	4.54%
Weighted Average	5.77%	5.60%	8.30%	7.00%	3.11%	−2.26%	5.84%
Meta-Learner using Statistics Average	5.55%	4.30%	7.94%	6.49%	2.04%	−3.72%	5.23%
Meta-Learner using probability							
Logistic Regression	3.33%	13.60%	4.80%	6.24%	7.37%	10.49%	5.39%
Random Forest	2.09%	11.80%	3.28%	2.20%	6.78%	7.26%	5.33%
AdaBoost	0.22%	11.60%	3.22%	4.58%	3.57%	8.75%	7.41%
Bagging Classifier SVC	2.42%	8.80%	4.72%	6.04%	5.59%	5.54%	8.80%
Bagging Classifier SGD	2.80%	9.20%	4.66%	5.16%	9.02%	6.99%	5.73%
Bagging Classifier LR	2.88%	13.30%	5.00%	5.98%	7.37%	10.53%	4.85%
GBM	2.77%	13.90%	4.69%	5.03%	6.65%	11.81%	8.89%
Gaussian NB	4.90%	16.70%	4.57%	4.80%	10.79%	15.38%	7.98%
Meta-Learner using Probability Average	2.68%	12.36%	4.37%	5.00%	7.14%	9.59%	6.80%
Max percentage	5.77%	16.70%	8.30%	7.00%	10.79%	15.38%	8.89%
Min percentage	0.22%	1.70%	3.22%	2.20%	0.73%	−5.06%	4.54%

## Data Availability

The MURA dataset underlying this study is a publicly available dataset available from arXiv:1712.06957.

## Appendix A

**Table A1:** The Kappa score achieved by each classifier of the test set for the humerus images (±Confidence Interval 95%). Test performance is reported.

	Accuracy	Kappa	AUC	Precision	Sensitivity	Specificity
Base-Learner						
VGG19	81.46% ± 1.22%	0.6299 ± 2.40%	81.57% ± 1.17%	78.49% ± 2.35%	77.57% ± 3.54%	85.57% ± 2.04%
InceptionV3	79.55% ± 1.39%	0.5918 ± 2.71%	79.66% ± 1.32%	76.73% ± 2.73%	75.54% ± 4.68%	83.79% ± 2.79%
ResNet50	76.67% ± 1.89%	0.5349 ± 3.67%	76.84% ± 1.80%	73.20% ± 3.21%	70.61% ± 5.82%	83.07% ± 3.32%
Xception	80.21% ± 1.30%	0.6045 ± 2.56%	80.27% ± 1.26%	78.29% ± 2.56%	78.04% ± 3.88%	82.50% ± 2.86%
DenseNet	78.68% ± 1.25%	0.5733 ± 2.50%	78.66% ± 1.25%	78.20% ± 1.90%	79.26% ± 2.72%	78.07% ± 2.62%
	Meta-Learner using Statistics					
Average	83.30% ± 0.81%	0.6662 ± 1.60%	83.36% ± 0.78%	81.27% ± 1.55%	81.28% ± 2.08%	85.43% ± 1.30%
Majority Vote	83.09% ± 0.84%	0.6621 ± 1.67%	83.15% ± 0.82%	80.93% ± 1.55%	80.88% ± 2.01%	85.43% ± 0.84%
Weighted Average	83.30% ± 0.86%	0.6662 ± 1.69%	83.35% ± 0.83%	81.31% ± 1.60%	81.35% ± 2.10%	85.36% ± 1.08%
	Meta-Learner using probability					
Logistic Regression	82.53% ± 0.66%	0.6508 ± 1.33%	82.58% ± 0.67%	80.78% ± 1.02%	81.01% ± 1.41%	84.14% ± 1.54%
RF Prop	82.15% ± 0.70%	0.6430 ± 1.40%	82.17% ± 0.69%	80.97% ± 1.18%	81.55% ± 1.48%	82.79% ± 0.92%
AdaBoost Prop	81.56% ± 0.79%	0.6312 ± 1.60%	81.59% ± 0.82%	80.20% ± 0.61%	80.74% ± 0.89%	82.43% ± 1.97%
Bagging Classifier SVC	82.26% ± 0.72%	0.6451 ± 1.44%	82.28% ± 0.73%	81.00% ± 0.87%	81.55% ± 1.12%	83.00% ± 1.50%
Bagging Classifier SGD	82.36% ± 0.55%	0.6475 ± 1.09%	82.42% ± 0.54%	80.24% ± 1.24%	80.20% ± 1.87%	84.64% ± 1.81%
Bagging Classifier LR	82.40% ± 0.59%	0.6480 ± 1.17%	82.44% ± 0.59%	80.73% ± 0.98%	81.01% ± 1.43%	83.86% ± 1.67%
GBM	82.36% ± 0.79%	0.6473 ± 1.59%	82.39% ± 0.80%	80.82% ± 0.96%	81.22% ± 1.24%	83.57% ± 1.60%
Gaussian NB	83.02% ± 0.65%	0.6607 ± 1.28%	83.09% ± 0.64%	80.74% ± 1.03%	80.68% ± 1.37%	85.50% ± 0.99%
	Meta-Learner using label					
Logistic Regression	82.08% ± 0.62%	0.6417 ± 1.25%	82.11% ± 0.63%	80.60% ± 0.92%	81.01% ± 1.30%	83.21% ± 1.57%
RF Label	81.60% ± 0.80%	0.6321 ± 1.60%	81.64% ± 0.79%	79.84% ± 1.25%	80.07% ± 1.63%	83.21% ± 1.28%
AdaBoost Label	82.01% ± 0.61%	0.6403 ± 1.22%	82.04% ± 0.61%	80.62% ± 0.99%	81.08% ± 1.40%	83.00% ± 1.67%
Bagging Classifier SVC	80.73% ± 1.21%	0.6145 ± 2.43%	80.74% ± 1.22%	79.59% ± 1.35%	80.27% ± 1.56%	81.21% ± 1.79%
Bagging Classifier SGD	81.01% ± 1.02%	0.6203 ± 2.04%	81.05% ± 1.02%	79.26% ± 1.50%	79.46% ± 2.00%	82.64% ± 1.82%
Bagging Classifier LR	82.05% ± 0.64%	0.6410 ± 1.28%	82.08% ± 0.64%	80.46% ± 1.03%	80.81% ± 1.41%	83.36% ± 1.43%
GBM	81.67% ± 0.92%	0.6335 ± 1.83%	81.72% ± 0.91%	79.80% ± 1.40%	79.93% ± 1.85%	83.50% ± 1.41%
Gaussian NB	82.29% ± 1.01%	0.6462 ± 2.01%	82.37% ± 1.01%	79.80% ± 1.14%	79.59% ± 1.34%	85.14% ± 1.27%

**Table A2:** The Kappa score achieved by each classifier for the finger images (±Confidence Interval 95%). Test performance is reported.

	Accuracy	Kappa	AUC	Precision	Sensitivity	Specificity
Base-Learner						
VGG19	67.77% ± 2.63%	0.3710 ± 4.83%	69.08% ± 2.41%	82.57% ± 2.69%	87.38% ± 2.94%	50.77% ± 6.05%
InceptionV3	67.25% ± 1.33%	0.3566 ± 2.41%	68.24% ± 1.17%	78.47% ± 3.26%	82.10% ± 4.73%	54.37% ± 5.47%
ResNet50	67.38% ± 1.82%	0.3605 ± 3.62%	68.49% ± 1.87%	79.80% ± 3.69%	84.11% ± 4.13%	52.87% ± 3.25%
Xception	69.74% ± 0.93%	0.4052 ± 1.68%	70.72% ± 0.82%	81.06% ± 1.85%	84.39% ± 2.74%	57.04% ± 3.45%
DenseNet	70.30% ± 1.90%	0.4168 ± 3.54%	71.31% ± 1.74%	82.15% ± 2.39%	85.42% ± 2.83%	57.21% ± 4.68%
	Meta-Learner using Statistics					
Average	71.41% ± 1.10%	0.4401 ± 2.01%	72.58% ± 0.98%	85.54% ± 1.11%	88.97% ± 1.33%	56.19% ± 2.81%
Majority Vote	70.54% ± 0.91%	0.4237 ± 1.68%	71.76% ± 0.83%	85.04% ± 1.55%	88.79% ± 1.60%	54.74% ± 2.45%
Weighted Average	71.41% ± 1.05%	0.4401 ± 1.92%	72.58% ± 0.94%	85.54% ± 1.12%	88.97% ± 1.33%	56.19% ± 2.71%
	Meta-Learner using probability					
Logistic Regression	73.36% ± 0.92%	0.4733 ± 1.83%	74.06% ± 0.93%	82.12% ± 1.42%	83.79% ± 1.59%	64.33% ± 1.35%
RF Prop	72.97% ± 1.06%	0.4660 ± 2.08%	73.71% ± 1.05%	82.09% ± 1.30%	83.79% ± 1.64%	62.63% ± 1.62%
AdaBoost Prop	72.97% ± 0.48%	0.4653 ± 0.98%	73.64% ± 0.52%	81.38% ± 1.26%	84.02% ± 1.31%	63.40% ± 1.37%
Bagging Classifier SVC	72.30% ± 0.81%	0.4536 ± 1.55%	73.11% ± 0.77%	82.15% ± 1.26%	82.90% ± 1.93%	63.20% ± 1.61%
Bagging Classifier SGD	72.39% ± 0.97%	0.4552 ± 1.81%	73.19% ± 0.88%	82.11% ± 1.07%	82.94% ± 1.55%	64.33% ± 1.03%
Bagging Classifier LR	73.32% ± 0.87%	0.4724 ± 1.72%	74.01% ± 0.88%	82.04% ± 1.39%	83.88% ± 1.71%	62.51% ± 1.75%
GBM	73.47% ± 0.76%	0.4748 ± 1.52%	74.10% ± 0.77%	81.56% ± 1.08%	84.44% ± 1.59%	61.78% ± 1.92%
Gaussian NB	74.12% ± 1.23%	0.4862 ± 2.42%	74.62% ± 1.22%	80.91% ± 1.44%	82.43% ± 2.07%	62.71% ± 1.57%
	Meta-Learner using label					
Logistic Regression	72.45% ± 0.84%	0.4560 ± 1.65%	73.21% ± 0.84%	81.73% ± 1.36%	83.79% ± 1.64%	62.63% ± 1.62%
RF Label	72.34% ± 0.95%	0.4533 ± 1.89%	73.05% ± 0.97%	81.08% ± 1.61%	84.02% ± 1.31%	63.40% ± 1.37%
AdaBoost Label	72.43% ± 0.83%	0.4556 ± 1.62%	73.19% ± 0.82%	81.80% ± 1.39%	82.90% ± 1.93%	63.20% ± 1.61%
Bagging Classifier SVC	71.87% ± 1.12%	0.4439 ± 2.22%	72.57% ± 1.14%	80.54% ± 1.82%	82.94% ± 1.55%	64.33% ± 1.03%
Bagging Classifier SGD	71.48% ± 1.43%	0.4367 ± 2.76%	72.23% ± 1.38%	80.50% ± 1.54%	83.88% ± 1.71%	62.51% ± 1.75%
Bagging Classifier LR	72.49% ± 0.86%	0.4568 ± 1.67%	73.25% ± 0.85%	81.75% ± 1.36%	84.44% ± 1.59%	61.78% ± 1.92%
GBM	72.30% ± 0.95%	0.4525 ± 1.89%	73.01% ± 0.98%	81.09% ± 1.68%	82.43% ± 2.07%	62.71% ± 1.57%
Gaussian NB	73.49% ± 1.15%	0.4718 ± 2.24%	73.80% ± 1.11%	78.58% ± 1.29%	84.35% ± 1.52%	62.02% ± 2.50%

**Table A3:** The Kappa score achieved by each classifier for the elbow images (±Confidence Interval 95%). Test performance is reported.

	Accuracy	Kappa	AUC	Precision	Sensitivity	Specificity
Base-Learner						
VGG19	82.21% ± 1.09%	0.6436 ± 2.19%	82.12% ± 1.10%	88.97% ± 2.06%	90.98% ± 2.01%	73.26% ± 2.70%
InceptionV3	80.62% ± 1.12%	0.6119 ± 2.25%	80.55% ± 1.12%	85.38% ± 2.37%	87.49% ± 2.62%	73.61% ± 2.16%
ResNet50	81.25% ± 1.08%	0.6243 ± 2.16%	81.16% ± 1.08%	86.95% ± 2.42%	89.06% ± 2.46%	73.26% ± 2.41%
Xception	82.19% ± 0.46%	0.6433 ± 0.91%	82.11% ± 0.46%	87.79% ± 2.26%	89.66% ± 2.32%	74.57% ± 2.29%
DenseNet	81.98% ± 1.05%	0.6390 ± 2.10%	81.91% ± 1.06%	86.74% ± 2.44%	88.47% ± 2.76%	75.35% ± 3.52%
	Meta-Learner using Statistics					
Average	84.83% ± 0.36%	0.6962 ± 0.73%	84.75% ± 0.37%	91.90% ± 0.92%	93.36% ± 0.85%	75.61% ± 1.45%
Majority Vote	84.58% ± 0.46%	0.6910 ± 0.94%	84.49% ± 0.47%	91.80% ± 0.87%	93.49% ± 0.90%	76.09% ± 1.29%
Weighted Average	84.88% ± 0.33%	0.6970 ± 0.67%	84.79% ± 0.34%	92.00% ± 0.92%	87.66% ± 1.23%	79.74% ± 1.29%
	Meta-Learner using probability					
Logistic Regression	83.74% ± 0.86%	0.6745 ± 1.71%	83.70% ± 0.86%	86.37% ± 1.17%	87.66% ± 1.23%	79.74% ± 1.29%
RF Prop	83.25% ± 1.02%	0.6647 ± 2.04%	83.21% ± 1.02%	85.65% ± 1.39%	86.94% ± 1.45%	79.48% ± 1.28%
AdaBoost Prop	83.23% ± 0.82%	0.6643 ± 1.64%	83.20% ± 0.82%	84.62% ± 1.01%	85.62% ± 1.09%	80.78% ± 1.27%
Bagging Classifier SVC	83.72% ± 0.74%	0.6740 ± 1.49%	83.67% ± 0.74%	87.29% ± 1.30%	88.77% ± 1.35%	78.57% ± 0.96%
Bagging Classifier SGD	83.70% ± 0.98%	0.6736 ± 1.97%	83.64% ± 0.98%	87.23% ± 1.64%	88.68% ± 1.66%	78.61% ± 1.50%
Bagging Classifier LR	83.81% ± 0.87%	0.6758 ± 1.74%	83.77% ± 0.87%	86.36% ± 1.16%	87.62% ± 1.22%	79.91% ± 1.33%
GBM	83.70% ± 0.81%	0.6738 ± 1.63%	83.67% ± 0.81%	85.25% ± 1.08%	86.26% ± 1.12%	81.09% ± 0.72%
Gaussian NB	83.66% ± 0.79%	0.6730 ± 1.58%	83.65% ± 0.79%	84.00% ± 0.92%	84.55% ± 1.01%	82.74% ± 1.21%
	Meta-Learner using label					
Logistic Regression	83.50% ± 0.81%	0.6699 ± 1.63%	83.47% ± 0.81%	85.59% ± 1.48%	86.72% ± 1.69%	80.22% ± 1.25%
RF Label	83.08% ± 0.93%	0.6612 ± 1.87%	83.03% ± 0.93%	85.93% ± 1.30%	87.36% ± 1.35%	78.70% ± 1.29%
AdaBoost Label	83.61% ± 0.89%	0.6720 ± 1.78%	83.58% ± 0.89%	85.53% ± 1.51%	86.60% ± 1.72%	80.57% ± 1.28%
Bagging Classifier SVC	82.84% ± 0.99%	0.6564 ± 1.98%	82.79% ± 0.99%	85.88% ± 1.79%	87.32% ± 1.95%	78.26% ± 1.69%
Bagging Classifier SGD	82.77% ± 1.06%	0.6551 ± 2.11%	82.73% ± 1.05%	85.69% ± 1.79%	87.11% ± 1.99%	78.35% ± 1.72%
Bagging Classifier LR	83.55% ± 0.76%	0.6707 ± 1.53%	83.51% ± 0.76%	85.91% ± 1.41%	87.11% ± 1.68%	79.91% ± 1.49%
GBM	83.27% ± 0.66%	0.6651 ± 1.32%	83.23% ± 0.66%	85.62% ± 1.01%	86.89% ± 1.18%	79.57% ± 1.41%
Gaussian NB	82.56% ± 1.21%	0.6512 ± 2.41%	82.57% ± 1.20%	81.74% ± 1.65%	81.70% ± 2.01%	83.43% ± 1.16%

**Table A4:** The Kappa score achieved by each classifier for the wrist images (±Confidence Interval 95%). Test performance is reported.

	Accuracy	Kappa	AUC	Precision	Sensitivity	Specificity
Base-Learner						
VGG19	80.53% ± 0.48%	0.6004 ± 0.99%	79.57% ± 0.52%	83.72% ± 2.11%	88.74% ± 2.10%	70.41% ± 2.49%
InceptionV3	80.02% ± 1.11%	0.5914 ± 2.16%	79.23% ± 1.00%	81.66% ± 2.80%	86.70% ± 2.82%	71.76% ± 2.14%
ResNet50	79.68% ± 1.18%	0.5830 ± 2.39%	78.73% ± 1.18%	82.63% ± 2.99%	87.86% ± 2.99%	69.59% ± 3.28%
Xception	81.15% ± 0.76%	0.6127 ± 1.59%	80.15% ± 0.82%	84.90% ± 1.94%	89.70% ± 1.84%	70.61% ± 2.50%
DenseNet	80.46% ± 0.86%	0.5984 ± 1.54%	79.44% ± 0.68%	84.63% ± 3.74%	89.18% ± 4.21%	69.69% ± 3.98%
	Meta-Learner using Statistics					
Average	83.25% ± 0.38%	0.6550 ± 0.72%	82.16% ± 0.32%	88.77% ± 1.78%	92.55% ± 1.51%	71.76% ± 1.38%
Majority Vote	82.87% ± 0.45%	0.6469 ± 0.86%	81.73% ± 0.39%	88.69% ± 1.87%	92.58% ± 1.57%	70.88% ± 1.38%
Weighted Average	83.28% ± 0.30%	0.6556 ± 0.58%	82.19% ± 0.26%	88.82% ± 1.59%	92.61% ± 1.34%	71.76% ± 1.30%
	Meta-Learner using probability					
Logistic Regression	82.97% ± 0.53%	0.6510 ± 1.06%	82.10% ± 0.51%	86.25% ± 1.36%	90.41% ± 1.26%	73.80% ± 1.25%
RF Prop	81.79% ± 0.68%	0.6262 ± 1.37%	80.84% ± 0.65%	85.23% ± 1.34%	89.95% ± 1.71%	73.22% ± 1.66%
AdaBoost Prop	82.49% ± 0.65%	0.6408 ± 1.33%	81.58% ± 0.66%	85.82% ± 0.97%	89.89% ± 1.06%	71.80% ± 0.73%
Bagging Classifier SVC	83.02% ± 0.43%	0.6497 ± 0.85%	81.85% ± 0.39%	89.23% ± 1.33%	91.51% ± 1.30%	72.03% ± 1.06%
Bagging Classifier SGD	82.69% ± 0.59%	0.6444 ± 1.16%	81.71% ± 0.54%	86.78% ± 1.67%	90.22% ± 0.74%	72.95% ± 0.93%
Bagging Classifier LR	82.90% ± 0.52%	0.6494 ± 1.04%	82.03% ± 0.50%	86.15% ± 1.31%	89.67% ± 1.78%	73.39% ± 1.63%
GBM	82.63% ± 0.53%	0.6436 ± 1.12%	81.72% ± 0.57%	86.03% ± 1.01%	93.05% ± 1.06%	70.64% ± 0.83%
Gaussian NB	82.50% ± 0.51%	0.6421 ± 1.04%	81.74% ± 0.51%	84.62% ± 0.96%	91.29% ± 2.02%	70.75% ± 1.37%
	Meta-Learner using label					
Logistic Regression	82.46% ± 0.58%	0.6404 ± 1.15%	81.58% ± 0.55%	85.63% ± 1.80%	89.95% ± 1.71%	73.22% ± 1.66%
RF Label	82.79% ± 0.59%	0.6462 ± 1.18%	81.77% ± 0.55%	87.38% ± 1.56%	89.89% ± 1.06%	71.80% ± 0.73%
AdaBoost Label	82.38% ± 0.60%	0.6390 ± 1.19%	81.53% ± 0.56%	85.33% ± 1.84%	91.51% ± 1.30%	72.03% ± 1.06%
Bagging Classifier SVC	82.09% ± 0.70%	0.6315 ± 1.35%	81.02% ± 0.59%	86.99% ± 2.36%	90.22% ± 0.74%	72.95% ± 0.93%
Bagging Classifier SGD	81.59% ± 0.92%	0.6225 ± 1.86%	80.69% ± 0.91%	84.57% ± 1.73%	89.67% ± 1.78%	73.39% ± 1.63%
Bagging Classifier LR	82.41% ± 0.49%	0.6394 ± 0.99%	81.53% ± 0.48%	85.59% ± 1.58%	93.05% ± 1.06%	70.64% ± 0.83%
GBM	82.64% ± 0.67%	0.6430 ± 1.33%	81.62% ± 0.61%	87.21% ± 1.74%	91.29% ± 2.02%	70.75% ± 1.37%
Gaussian NB	81.52% ± 0.80%	0.6236 ± 1.59%	80.96% ± 0.76%	81.79% ± 1.76%	90.99% ± 1.43%	72.44% ± 1.07%

**Table A5:** The Kappa score achieved by each classifier for the forearm images (±Confidence Interval 95%). Test performance is reported.

	Accuracy	Kappa	AUC	Precision	Sensitivity	Specificity
Base-Learner						
VGG19	75.38% ± 1.27%	0.5081 ± 2.55%	75.43% ± 1.28%	85.94% ± 3.50%	89.53% ± 3.48%	61.32% ± 2.78%
InceptionV3	75.12% ± 2.19%	0.5028 ± 4.36%	75.16% ± 2.18%	85.23% ± 2.59%	89.40% ± 1.79%	60.93% ± 3.19%
ResNet50	77.14% ± 1.82%	0.5432 ± 3.64%	77.18% ± 1.82%	86.83% ± 4.25%	89.73% ± 3.90%	64.64% ± 2.11%
Xception	76.35% ± 1.56%	0.5274 ± 3.11%	76.40% ± 1.55%	89.73% ± 1.67%	93.00% ± 1.53%	59.80% ± 3.98%
DenseNet	77.94% ± 1.08%	0.5592 ± 2.16%	77.98% ± 1.08%	87.54% ± 2.14%	90.47% ± 2.04%	65.50% ± 2.91%
	Meta-Learner using statistics					
Average	78.57% ± 1.02%	0.5719 ± 2.04%	78.63% ± 1.02%	92.73% ± 0.85%	94.47% ± 0.78%	61.92% ± 2.29%
Majority Vote	78.14% ± 1.09%	0.5633 ± 2.18%	78.19% ± 1.09%	91.87% ± 1.02%	95.00% ± 0.75%	62.72% ± 2.29%
Weighted Average	78.80% ± 0.93%	0.5765 ± 1.85%	78.86% ± 0.93%	92.71% ± 0.88%	93.93% ± 1.18%	66.16% ± 2.39%
	Meta-Learner using probability					
Logistic Regression	80.00% ± 0.88%	0.6004 ± 1.76%	80.05% ± 0.88%	91.74% ± 1.30%	93.93% ± 1.18%	66.16% ± 2.39%
RF Prop	79.83% ± 1.19%	0.5971 ± 2.38%	79.88% ± 1.19%	91.70% ± 1.48%	94.00% ± 1.10%	65.76% ± 1.79%
AdaBoost Prop	78.94% ± 0.96%	0.5791 ± 1.91%	78.98% ± 0.96%	89.85% ± 1.27%	92.53% ± 1.07%	65.43% ± 1.76%
Bagging Classifier SVC	79.50% ± 1.32%	0.5904 ± 2.64%	79.55% ± 1.32%	92.00% ± 1.56%	94.27% ± 1.30%	64.83% ± 2.88%
Bagging Classifier SGD	80.47% ± 0.98%	0.6096 ± 1.96%	80.50% ± 0.98%	90.21% ± 1.58%	92.40% ± 1.62%	68.61% ± 2.82%
Bagging Classifier LR	80.00% ± 1.04%	0.6004 ± 2.08%	80.05% ± 1.04%	91.89% ± 1.30%	94.07% ± 1.16%	66.03% ± 2.70%
GBM	79.80% ± 1.14%	0.5964 ± 2.28%	79.84% ± 1.14%	90.08% ± 1.31%	92.53% ± 1.12%	67.15% ± 2.14%
Gaussian NB	80.96% ± 1.00%	0.6195 ± 2.00%	81.00% ± 1.00%	89.03% ± 1.18%	91.20% ± 1.05%	70.79% ± 1.71%
	Meta-Learner using label					
Logistic Regression	79.47% ± 0.97%	0.5897 ± 1.93%	79.51% ± 0.96%	90.75% ± 1.01%	93.20% ± 0.97%	65.83% ± 2.49%
RF Label	79.93% ± 1.21%	0.5990 ± 2.42%	79.98% ± 1.21%	90.33% ± 1.45%	92.67% ± 1.35%	67.28% ± 3.04%
AdaBoost Label	79.44% ± 0.93%	0.5891 ± 1.85%	79.48% ± 0.92%	90.26% ± 1.80%	92.67% ± 1.87%	66.29% ± 2.78%
Bagging Classifier SVC	78.97% ± 1.43%	0.5797 ± 2.87%	79.01% ± 1.43%	88.57% ± 2.71%	91.07% ± 2.76%	66.95% ± 3.39%
Bagging Classifier SGD	79.53% ± 1.31%	0.5910 ± 2.62%	79.57% ± 1.31%	88.39% ± 2.50%	90.73% ± 2.60%	68.41% ± 3.44%
Bagging Classifier LR	79.30% ± 0.88%	0.5864 ± 1.76%	79.35% ± 0.88%	90.11% ± 1.91%	92.53% ± 2.01%	66.16% ± 2.90%
GBM	80.10% ± 0.84%	0.6023 ± 1.68%	80.14% ± 0.84%	90.60% ± 1.51%	92.87% ± 1.42%	67.42% ± 2.55%
Gaussian NB	81.00% ± 0.88%	0.6201 ± 1.76%	81.02% ± 0.88%	87.05% ± 0.76%	89.07% ± 0.75%	72.98% ± 1.81%

**Table A6:** The Kappa score achieved by each classifier for the hand images (±Confidence Interval 95%). Test performance is reported.

	Accuracy	Kappa	AUC	Precision	Sensitivity	Specificity
Base-Learner						
VGG19	74.59% ± 0.69%	0.4358 ± 1.68%	70.28% ± 0.84%	85.33% ± 1.65%	94.43% ± 0.83%	46.14% ± 1.98%
InceptionV3	70.93% ± 1.13%	0.3473 ± 3.01%	66.00% ± 1.50%	81.40% ± 2.99%	93.69% ± 1.63%	38.31% ± 4.0%
ResNet50	71.37% ± 1.67%	0.3603 ± 4.84%	66.76% ± 2.49%	80.66% ± 3.51%	92.62% ± 2.88%	40.90% ± 7.45%
Xception	73.17% ± 1.60%	0.3940 ± 4.39%	68.09% ± 2.27%	90.08% ± 3.23%	96.61% ± 1.9%	39.58% ± 6.18%
DenseNet	72.48% ± 1.58%	0.3810 ± 4.06%	67.54% ± 2.01%	85.56% ± 2.44%	95.24% ± 1.12%	39.84% ± 4.55%
	Meta-Learner using statistics					
Average	74.17% ± 1.02%	0.4190 ± 2.60%	69.26% ± 1.31%	88.02% ± 4.54%	96.83% ± 0.43%	41.69% ± 2.95%
Majority Vote	73.98% ± 1.04%	0.4138 ± 2.63%	68.99% ± 1.31%	90.48% ± 1.16%	96.97% ± 0.52%	41.01% ± 2.88%
Weighted Average	74.43% ± 0.91%	0.4260 ± 2.30%	69.61% ± 1.16%	89.98% ± 1.02%	96.68% ± 0.47%	42.54% ± 2.57%
	Meta-Learner using probability					
Logistic Regression	76.43% ± 0.73%	0.4815 ± 1.56%	72.60% ± 0.72%	85.87% ± 2.11%	94.10% ± 1.01%	51.11% ± 0.95%
RF Prop	75.72% ± 0.89%	0.4675 ± 2.00%	72.02% ± 0.97%	83.23% ± 1.91%	94.58% ± 1.06%	48.52% ± 1.58%
AdaBoost Prop	75.98% ± 0.48%	0.4740 ± 0.99%	72.35% ± 0.45%	83.33% ± 1.78%	92.77% ± 0.97%	51.27% ± 1.72%
Bagging Classifier SVC	75.65% ± 0.87%	0.4600 ± 1.93%	71.42% ± 0.91%	87.44% ± 2.58%	94.46% ± 0.87%	49.58% ± 1.75%
Bagging Classifier SGD	75.85% ± 1.16%	0.4663 ± 2.58%	71.79% ± 1.21%	86.34% ± 2.91%	92.69% ± 1.01%	52.01% ± 1.01%
Bagging Classifier LR	76.43% ± 0.72%	0.4817 ± 1.52%	72.62% ± 0.70%	85.75% ± 2.12%	94.61% ± 1.04%	48.52% ± 1.58%
GBM	76.57% ± 0.81%	0.4873 ± 1.86%	73.01% ± 0.93%	84.04% ± 1.46%	95.17% ± 1.16%	47.67% ± 1.58%
Gaussian NB	76.96% ± 0.57%	0.5029 ± 1.14%	74.07% ± 0.51%	80.63% ± 1.66%	94.91% ± 0.85%	48.10% ± 1.91%
	Meta-Learner using label					
Logistic Regression	75.65% ± 0.63%	0.4614 ± 1.42%	71.55% ± 0.69%	86.33% ± 2.21%	94.58% ± 1.06%	48.52% ± 1.58%
RF Label	76.02% ± 0.62%	0.4706 ± 1.48%	72.02% ± 0.74%	86.30% ± 1.72%	92.77% ± 0.97%	51.27% ± 1.72%
AdaBoost Label	75.67% ± 0.59%	0.4619 ± 1.35%	71.57% ± 0.66%	86.41% ± 2.13%	94.46% ± 0.87%	49.58% ± 1.75%
Bagging Classifier SVC	75.67% ± 0.87%	0.4611 ± 2.02%	71.50% ± 0.99%	86.89% ± 1.94%	92.69% ± 1.01%	52.01% ± 1.01%
Bagging Classifier SGD	75.85% ± 0.74%	0.4659 ± 1.73%	71.77% ± 0.86%	86.62% ± 2.28%	94.61% ± 1.04%	48.52% ± 1.58%
Bagging Classifier LR	75.70% ± 0.66%	0.4626 ± 1.51%	71.61% ± 0.74%	86.29% ± 2.23%	95.17% ± 1.16%	47.67% ± 1.58%
GBM	76.15% ± 0.62%	0.4739 ± 1.47%	72.19% ± 0.74%	86.32% ± 1.83%	94.91% ± 0.85%	48.10% ± 1.91%
Gaussian NB	76.37% ± 1.29%	0.4962 ± 2.62%	74.03% ± 1.22%	76.96% ± 2.77%	94.54% ± 1.29%	49.05% ± 1.78%

**Table A7:** The Kappa score achieved by each classifier for the shoulder images (±Confidence Interval 95%). Test performance is reported.

	Accuracy	Kappa	AUC	Precision	Sensitivity	Specificity
Base-Learner						
VGG19	73.21% ± 1.26%	0.4638 ± 2.49%	73.17% ± 1.23%	75.09% ± 3.09%	77.12% ± 4.49%	69.21% ± 3.18%
InceptionV3	69.38% ± 2.18%	0.3888 ± 4.35%	69.48% ± 2.18%	66.27% ± 2.13%	61.23% ± 4.10%	77.73% ± 3.64%
ResNet50	70.67% ± 2.30%	0.4145 ± 4.53%	70.76% ± 2.25%	68.19% ± 3.61%	63.89% ± 6.53%	77.63% ± 2.52%
Xception	70.98% ± 1.73%	0.4207 ± 3.43%	71.08% ± 1.71%	67.78% ± 2.23%	63.12% ± 3.81%	79.03% ± 1.95%
DenseNet	72.61% ± 1.39%	0.4527 ± 2.73%	72.66% ± 1.35%	70.90% ± 2.83%	68.88% ± 4.99%	76.44% ± 3.10%
	Meta-Learner using statistics					
Average	74.39% ± 1.10%	0.4884 ± 2.17%	74.45% ± 1.08%	71.69% ± 1.66%	69.12% ± 2.85%	79.78% ± 1.71%
Majority Vote	74.21% ± 1.29%	0.4848 ± 2.57%	74.27% ± 1.28%	71.73% ± 1.88%	69.40% ± 3.19%	79.14% ± 1.88%
Weighted Average	74.51% ± 1.10%	0.4908 ± 2.19%	74.57% ± 1.09%	71.94% ± 1.69%	69.58% ± 2.89%	79.57% ± 1.66%
	Meta-Learner using probability					
Logistic Regression	74.42% ± 1.32%	0.4887 ± 2.63%	74.45% ± 1.31%	72.87% ± 1.54%	72.04% ± 1.95%	76.87% ± 0.95%
RF Prop	74.44% ± 0.82%	0.4885 ± 1.64%	74.41% ± 0.82%	75.14% ± 0.97%	76.70% ± 1.17%	72.12% ± 1.24%
AdaBoost Prop	74.90% ± 1.28%	0.4981 ± 2.56%	74.91% ± 1.28%	74.14% ± 1.62%	74.21% ± 2.07%	75.61% ± 1.46%
Bagging Classifier SVC	75.24% ± 1.21%	0.5045 ± 2.42%	75.22% ± 1.20%	75.75% ± 1.75%	76.98% ± 2.12%	73.45% ± 0.88%
Bagging Classifier SGD	74.51% ± 1.80%	0.4903 ± 3.58%	74.52% ± 1.78%	73.85% ± 2.79%	73.68% ± 3.94%	75.36% ± 1.23%
Bagging Classifier LR	74.30% ± 1.30%	0.4863 ± 2.58%	74.33% ± 1.29%	72.70% ± 1.57%	71.79% ± 2.05%	76.87% ± 0.96%
GBM	75.26% ± 1.05%	0.5050 ± 2.09%	75.24% ± 1.04%	75.42% ± 1.28%	76.42% ± 1.48%	74.06% ± 1.11%
Gaussian NB	75.03% ± 0.88%	0.5008 ± 1.75%	75.05% ± 0.87%	73.59% ± 1.14%	72.95% ± 1.59%	77.16% ± 0.93%
	Meta-Learner using label					
Logistic Regression	74.17% ± 1.26%	0.4839 ± 2.50%	74.22% ± 1.24%	72.17% ± 1.93%	70.56% ± 3.00%	77.91% ± 1.24%
RF Label	74.19% ± 1.24%	0.4839 ± 2.45%	74.20% ± 1.22%	73.72% ± 2.12%	73.02% ± 2.93%	74.78% ± 1.17%
AdaBoost Label	73.00% ± 2.14%	0.4606 ± 4.24%	73.05% ± 2.10%	71.33% ± 3.43%	70.54% ± 3.03%	77.91% ± 1.24%
Bagging Classifier SVC	74.30% ± 1.31%	0.4863 ± 2.60%	74.34% ± 1.29%	72.51% ± 1.75%	73.89% ± 3.07%	74.50% ± 1.63%
Bagging Classifier SGD	74.05% ± 1.23%	0.4810 ± 2.45%	74.05% ± 1.22%	73.54% ± 1.95%	69.33% ± 5.41%	76.76% ± 1.90%
Bagging Classifier LR	74.39% ± 1.15%	0.4882 ± 2.30%	74.43% ± 1.15%	72.39% ± 1.40%	71.26% ± 2.55%	77.41% ± 0.96%
GBM	74.17% ± 1.26%	0.4839 ± 2.50%	74.22% ± 1.24%	72.17% ± 1.93%	73.75% ± 2.78%	74.35% ± 1.51%
Gaussian NB	74.19% ± 1.24%	0.4839 ± 2.45%	74.20% ± 1.22%	73.72% ± 2.12%	70.95% ± 2.19%	77.91% ± 2.04%

**Table A8:** The percentage difference between the Kappa score of the highest level-0 classifiers (CNNs) to the Kappa score of the level-1 classifiers (machine learning algorithms). Test performance is reported.

	Humerus	Finger	Elbow	Wrist	Forearm	Hand	Shoulder
Reference	VGG19	DenseNet121	VGG19	Xception	DenseNet121	VGG19	VGG19
Meta-Learner using Statistics							
Average	5.77%	5.60%	8.17%	6.90%	2.28%	−3.85%	5.31%
Majority Vote	5.12%	1.70%	7.36%	5.58%	0.73%	−5.06%	4.54%
Weighted Average	5.77%	5.60%	8.30%	7.00%	3.11%	−2.26%	5.84%
Meta-Learner using Statistics Average	5.55%	4.30%	7.94%	6.49%	2.04%	−3.72%	5.23%
Meta-Learner using probability							
Logistic Regression	3.33%	13.60%	4.80%	6.24%	7.37%	10.49%	5.39%
RF Prop	2.09%	11.80%	3.28%	2.20%	6.78%	7.26%	5.33%
AdaBoost Prop	0.22%	11.60%	3.22%	4.58%	3.57%	8.75%	7.41%
Bagging Classifier SVC Prop	2.42%	8.80%	4.72%	6.04%	5.59%	5.54%	8.80%
Bagging Classifier SGD Prop	2.80%	9.20%	4.66%	5.16%	9.02%	6.99%	5.73%
Bagging Classifier LR Prop	2.88%	13.30%	5.00%	5.98%	7.37%	10.53%	4.85%
GBM Prop	2.77%	13.90%	4.69%	5.03%	6.65%	11.81%	8.89%
Gaussian NB Prop	4.90%	16.70%	4.57%	4.80%	10.79%	15.38%	7.98%
Meta-Learner using Probability Average	2.68%	12.36%	4.37%	5.00%	7.14%	9.59%	6.80%
Meta-Learner using label							
Logistic Regression	1.88%	9.40%	4.09%	4.51%	5.47%	5.88%	4.43%
RF Label	0.36%	8.80%	2.73%	5.46%	7.13%	7.98%	3.05%
AdaBoost Label	1.66%	9.30%	4.41%	4.29%	5.35%	5.97%	4.35%
Bagging Classifier SVC Label	−2.44%	6.50%	1.99%	3.06%	3.68%	5.79%	4.33%
Bagging Classifier SGD Label	−1.52%	4.80%	1.79%	1.60%	5.69%	6.91%	−0.69%
Bagging Classifier LR Label	1.78%	9.60%	4.21%	4.35%	4.87%	6.14%	4.87%
GBM Label	0.58%	8.60%	3.34%	4.95%	7.72%	8.73%	3.72%
Gaussian NB Label	2.60%	13.20%	1.18%	1.78%	10.90%	13.86%	5.26%
Meta-Learner using label Average	0.61%	8.78%	2.97%	3.75%	6.35%	7.66%	3.67%
Max percentage	5.77%	16.70%	8.30%	7.00%	10.90%	15.38%	8.89%
Min percentage	−2.44%	1.70%	1.18%	1.60%	0.73%	−5.06%	−0.69%

**Table A9:** The Average Training Time.

	Humerus	Finger	Elbow	Wrist	Forearm	Hand	Shoulder
Meta-Learner using Statistics							
VGG19	8 Min	40 Min	53 Min	103 Min	18 Min	86 Min	109 Min
InceptionV3	11 Min	32 Min	39 Min	91 Min	15 Min	64 Min	71 Min
ResNet50	9 Min	50 Min	48 Min	106 Min	20 Min	61 Min	69 Min
Xception	8 Min	38 Min	42 Min	73 Min	18 Min	41 Min	67 Min
DenseNet	10 Min	56 Min	47 Min	92 Min	18 Min	58 Min	78 Min
Meta-Learner using probability							
Logistic Regression	2 S	2 S	2 S	2 S	2 S	2 S	2 S
RF Prop	2 S	2 S	2 S	2 S	2 S	2 S	2 S
AdaBoost Prop	2 S	2 S	2 S	2 S	2 S	2 S	2 S
Bagging Classifier SVC Prop	2 S	2 S	2 S	2 S	2 S	2 S	2 S
Bagging Classifier SGD Prop	2 S	2 S	2 S	2 S	2 S	2 S	2 S
Bagging Classifier LR Prop	2 S	2 S	2 S	2 S	2 S	2 S	2 S
GBM Prop	2 S	2 S	2 S	2 S	2 S	2 S	2 S
Gaussian NB Prop	2 S	2 S	2 S	2 S	2 S	2 S	2 S
Meta-Learner using label							
Logistic Regression	2 S	2 S	2 S	2 S	2 S	2 S	2 S
RF Label	2 S	2 S	2 S	2 S	2 S	2 S	2 S
AdaBoost Label	2 S	2 S	2 S	2 S	2 S	2 S	2 S
Bagging Classifier SVC Label	2 S	2 S	2 S	2 S	2 S	2 S	2 S
Bagging Classifier SGD Label	2 S	2 S	2 S	2 S	2 S	2 S	2 S
Bagging Classifier LR Label	2 S	2 S	2 S	2 S	2 S	2 S	2 S
GBM Label	2 S	2 S	2 S	2 S	2 S	2 S	2 S
Gaussian NB Label	2 S	2 S	2 S	2 S	2 S	2 S	2 S

**Table A10:** The Average Testing Time.

	Humerus	Finger	Elbow	Wrist	Forearm	Hand	Shoulder
Meta-Learner using Statistics							
VGG19	8 S	9 S	11 S	15 S	7 S	9 S	12 S
InceptionV3	6 S	9 S	9 S	14 S	7 S	13 S	10 S
ResNet50	7 S	12 S	12 S	14 S	7 S	10 S	9 S
Xception	8 S	9 S	10 S	12 S	6 S	10 S	9 S
DenseNet	14 S	12 S	18 S	19 S	10 S	17 S	12 S
Meta-Learner using probability							
Logistic Regression	1 S	1 S	1 S	1 S	1 S	1 S	1 S
RF Prop	1 S	1 S	1 S	1 S	1 S	1 S	1 S
AdaBoost Prop	1 S	1 S	1 S	1 S	1 S	1 S	1 S
Bagging Classifier SVC Prop	1 S	1 S	1 S	1 S	1 S	1 S	1 S
Bagging Classifier SGD Prop	1 S	1 S	1 S	1 S	1 S	1 S	1 S
Bagging Classifier LR Prop	1 S	1 S	1 S	1 S	1 S	1 S	1 S
GBM Prop	1 S	1 S	1 S	1 S	1 S	1 S	1 S
Gaussian NB Prop	1 S	1 S	1 S	1 S	1 S	1 S	1 S
Meta-Learner using label							
Logistic Regression	1 S	1 S	1 S	1 S	1 S	1 S	1 S
RF Label	1 S	1 S	1 S	1 S	1 S	1 S	1 S
AdaBoost Label	1 S	1 S	1 S	1 S	1 S	1 S	1 S
Bagging Classifier SVC Label	1 S	1 S	1 S	1 S	1 S	1 S	1 S
Bagging Classifier SGD Label	1 S	1 S	1 S	1 S	1 S	1 S	1 S
Bagging Classifier LR Label	1 S	1 S	1 S	1 S	1 S	1 S	1 S
GBM Label	1 S	1 S	1 S	1 S	1 S	1 S	1 S
Gaussian NB Label	1 S	1 S	1 S	1 S	1 S	1 S	1 S
